# GWAS-identified hyperuricemia-associated *IGF1R* variant rs6598541 has a limited role in urate mediated inflammation in human mononuclear cells

**DOI:** 10.1038/s41598-024-53209-7

**Published:** 2024-02-12

**Authors:** Orsolya I. Gaal, Ruiqi Liu, Dragoș Marginean, Medeea Badii, Georgiana Cabău, Ioana Hotea, Valentin Nica, Doina Colcear, HINT Consortium, Leo A. B. Joosten, Ioan V. Pop, Tania O. Crişan, Marius Farcaş, Dragoş H. Marginean, Medeea O. Badii, Loredana Peca, Andreea-Manuela Mirea, Mariana S. Pop, Ancuta Rus, Cristina Pamfil, Tony R. Merriman, Simona Rednic, Radu A. Popp, Tania O. Crișan, Leo A. B. Joosten

**Affiliations:** 1 Department of Medical Genetics, Iuliu Hațieganu University of Medicine and Pharmacy, Str. Pasteur Nr.6, 400349 Cluj-Napoca, Romania; 2 Department of Internal Medicine, Radboud University Medical Center, Nijmegen, The Netherlands; 3 Department of Rheumatology, Iuliu Hațieganu University of Medicine and Pharmacy, Cluj-Napoca, Romania; 4 Department of Microbiology, University of Otago, Dunedin, New Zealand; 5 Division of Clinical Immunology and Rheumatology, University of Alabama at Birmingham, Birmingham, AL USA; 6 Clinical Infectious Disease Hospital, Cluj-Napoca, Romania

**Keywords:** Functional genomics, Gene expression, Genetic association study, Genetic markers, Inflammation

## Abstract

Gout is a common autoinflammatory joint diseases characterized by deposition of monosodium urate (MSU) crystals which trigger an innate immune response mediated by inflammatory cytokines. *IGF1R* is one of the loci associated with both urate levels and gout susceptibility in GWAS to date, and IGF-1-IGF-1R signaling is implicated in urate control. We investigate the role of IGF-1/IGF1R signaling in the context of gouty inflammation. Also, we test the gout and urate-associated *IGF1R* rs6598541 polymorphism for association with the inflammatory capacity of mononuclear cells. For this, freshly isolated human peripheral blood mononuclear cells (PBMCs) were exposed to recombinant IGF-1 or anti-IGF1R neutralizing antibody in the presence or absence of solubilized urate, stimulated with LPS/MSU crystals. Also, the association of rs6598541 with *IGF1R* and protein expression and with ex vivo cytokine production levels after stimulation with gout specific stimuli was tested. Urate exposure was not associated with *IGF1R* expression in vitro or in vivo. Modulation of IGF1R did not alter urate-induced inflammation. Developing urate-induced trained immunity in vitro was not influenced in cells challenged with IGF-1 recombinant protein. Moreover, the *IGF1R* rs6598541 SNP was not associated with cytokine production. Our results indicate that urate-induced inflammatory priming is not regulated by IGF-1/IGF1R signaling in vitro. *IGF1R* rs6598541 status was not asociated with *IGF1R* expression or cytokine production in primary human PBMCs. This study suggests that the role of IGF1R in gout is tissue-specific and may be more relevant in the control of urate levels rather than in inflammatory signaling in gout.

## Introduction

Gout is an important inflammatory disease with high prevalence in developed countries among men and postmenopausal women^1,2^ with prevalence increasing worldwide^3^. The precondition for developing gout is the deposition of monosodium urate (MSU) crystals in the joint and other tissues as a result of elevated serum urate levels (hyperuricemia)^4^. While most research focuses on inflammation due to MSU crystal deposition^5–7^, there is evidence that soluble urate also increases pro-inflammatory cytokine production^8^, associating hyperuricemia with a hyper-inflammatory state^9,10^, highlighting its pro-inflammatory effects within the intracellular setting together with an altered epigenetic landscape^11^. Additionally, there exist indication on how soluble urate induce epigenetic modifications in myeloid cells, leading to an enhanced inflammatory response^12,13^. Moreover, hyperuricemia may lead to innate immune memory, contributing to a persistently elevated inflammatory status^11,12,14^. In conditions associated with chronic inflammation, such as metabolic syndrome, obesity and cardiovascular disease, there is an increased prevalence of hyperuricemia, suggesting that the link between hyperuricemia and inflammation is perhaps bidirectional. While inflammation itself may influence urate levels, alternatively elevated urate levels may add to an inflammatory state. The pathogenetic mechanism of gout at molecular level is not well established. Although, several treatment possibilities are already available such as colchicine, nonsteroidal anti-inflammatory drugs, urate lowering therapies, and anti-IL-1 therapies. Nevertheless, potential side effects of therapy are also worth noting, including hypersensitivity, poor tolerance or risk of infection, therefore, finding other effective and better tolerated categories of modulators would be a great benefit for gout patients.

IGF-1 is an important growth factor with signaling roles in numerous cell types, including monocytes^15^, macrophages^16^ and chondrocytes^17^. The protein binds with high affinity to the Insulin Like Growth Factor 1 Receptor (IGF1R)^18^. The activated receptor is engaged in cell growth, survival control as well as proliferation and is also known for being involved in metabolic regulation^19,20^. Previous genome-wide association studies on serum urate concentrations^21,22^, identified *IGF1R* as a genetic locus for serum urate levels. The index SNP with the lowest *p* value at the *IGF1R* locus in the study performed by Köttgen et al*.* was rs6598541, of which the minor allele “A” was associated with increased urate levels (0.043 mg/dl increase [CI 0.031–0.055], p = 5 × 10^−15^)^21^. This variant also associated with urate control in a trans-ancestral meta-analysis study in European and East Asian populations^23^. Moreover, this SNP also associated with gout in patients that met the American College of Rheumatology (ACR) classification criteria for gout^24^.

The IGF-1 pathway was recently linked to innate immune memory and proinflammatory reprogramming induced by metabolic stimuli in human primary monocytes^25^. Stimulation via the IGF1R by mevalonate or by IGF-1 itself was able to induce trained immunity and epigenetic modifications in human primary monocytes and this contributed to enhanced proinflammatory responses to subsequent stimulation with LPS or Pam3Cys^25^. SNPs in *IGF1R* were significantly associated to cytokine production in response to training with BCG or β-glucan in vitro^25^*.* Additional evidence shows IGF-1 as having pro-inflammatory effects upon 24 h stimulation in primary human PBMCs, albeit these effects were only visible in synergy with TLR ligands such as LPS or Pam3Cys^26^. These effects were reported to be mediated via the MAPK pathway^26^. Other evidence shows that IGF-1 enhances chemotactic macrophage migration which leads to tissue inflammation^27^.

In the present study we address the hypothesis that signaling via the IGF1R is associated to inflammation in gout. We investigated whether soluble urate modulates *IGF1R* gene expression in human cells and whether the IGF-1 pathway has an effect on the proinflammatory priming elicited by soluble urate in vitro. Moreover, we assessed the association of the *IGF1R* rs6598541 SNP with *IGF1R* expression in circulating mononuclear cells and cytokine production capacity in patients with gout, asymptomatic hyperuricemia and normouricemic controls in an Eastern European population.

## Materials and methods

### Participants

The participants in this study consisted of patients with gout (n = 116), hyperuricemic controls (n = 78) and normouricemic volunteers (n = 174), followed at the Rheumatology Department of the “Iuliu Haţieganu” University of Medicine and Pharmacy, Cluj-Napoca, Romania. Subjects were enrolled after written informed consent. Peripheral blood was drawn from the cubital vein on EDTA tubes under sterile conditions. The patient study was approved by the Ethical Committee of the “Iuliu Hațieganu” University of Medicine and Pharmacy, Cluj-Napoca (approval no. 425/2016) and all participants provided written informed consent. Experiments were conducted according to the principles expressed in the Declaration of Helsinki. All study participants in the gout group were included if they corresponded to the ACR/EULAR 2015 classification criteria with a score of $\ge$ 8. The cutoff for the asyptomatic hyperuricemia group was a serum urate concentration of 7 mg/dl and negative history of gout flares. The described groups were similar in age and BMI. The gender distribution shows a higher number of men in the gout study group, consistent with the higher prevalence of gout in males.

### PBMC isolation and stimulation

Isolation and stimulation of human peripheral blood mononuclear cells (PBMCs) was assessed as described previously^8^. Briefly, PBMCs were separated using Ficoll-Paque and resuspended in RPMI culture medium with Dutch modification (Gibco), supplemented with human pooled serum. Monocyte isolation was done using hyperosmotic Percoll^®^ solution^28^. Cells were incubated for 24 h with culture medium as negative control, and urate in different concentrations. After priming, culture medium was removed, the remaining adherent cells were washed with warm PBS, then restimulated with medium or LPS with or without MSU crystals. IGF1R was induced by the addition of IGF-1 (R&D Systems, Abingdon, United Kingdom) in concentration of 5 μg/ml. In separate experiments IGF1R signaling was blocked with anti-IGF1R antibody (R&D Systems).

### Cytokine measurements

Cytokine concentrations were determined in cell culture supernatants using specific sandwich ELISA kits for IL-1β, IL-1Ra, IL-6 (R&D Systems, Minneapolis).

### Qantitative PCR for mRNA expression of *IGF1R*

PBMCs were incubated with the stimuli as mentioned above and after 24 h of incubation at 37 °C in 5% CO_2_. The plates were centrifuged, the supernatant was collected and the cell pellets were lysed with 300 μl of TRIzol Reagent (Invitrogen). RNA purifcation was performed according to manufacturer’s instructions. Subsequently, RNA concentrations were determined using NanoDrop software. Isolated RNA was subsequently transcribed into complementary DNA using High-Capacity cDNA Reverse Transcription Kit (Applied BioSystems) followed by quantitative PCR using the Sybr Green Method. The following primers were used in the reaction: *IGF1R* forward 5′-TCGACATCCGCAACGACTATC-3′ and reverse 5′-TCGACATCCGCAACGACTATC-3′ and β2-microglobulin forward 5′-ATGAGTATGCCTGCCGTGTG-3′ and reverse 5′-CCAAATGCGGCATCTTCAAAC-3′. Results are shown as fold change in mRNA levels in stimulated samples compared to controls.

### Genotyping for *IGF1R* rs6598541

Three independent groups were genotyped (gout group N = 116; hyperuricemic group N = 78 and healthy volunteers group N = 174). Genomic DNA was isolated from whole blood (Promega) and genotyping was performed on an Illumina Infinium HD assay platform using The Infinium Global Screening Array-24 v3.0 BeadChip. The quality control protocol for genotyping data was performed Using Illumina's GenomeStudio. The SNPs with < 95% call rate were excluded and all the SNPs with 95–98% call rate were verified and manually re-clustered or removed when necessary. The data was exported to PLINK format and further filters were applied: minor allele frequency > 0.01; hardy–weinberg equilibrium test p value > 10^–6^; samples with heterozygosity rate of ± 3 standard deviations and related individuals were excluded. For the final step, the strands were flipped and all the data was verified to align to the GRCh37 hg19 build. The genotypes generated were checked using a predesigned TaqMan SNP genotyping Assay (Applied Biosystems).

### Transcriptomics

Freshly isolated PBMCs were frozen in TRIzol Reagent (Invitrogen) and stored at − 80 °C and were later used for commercial RNA-Seq analysis (Beijing Genomics Institute, BGI, Beijing, China). The integrity of extracted RNA was assessed using Agilent 2100 Bioanalyzer. Oligo dT magnetic beads were used to capture mRNA from total RNA. Purifed ligation products were enriched using PCR amplifcation followed by denaturation and cyclization of ssDNA by splint oligos and DNA ligase generating DNA nanoballs (DNBs). Sequencing of DNBs was performed on DNBseq platform.

Initial quality control was performed with SOAPnuke (v1.5.2). Clean reads were mapped to human transcriptome assembly GRCh37 (hg19) using bowtie2. Read counts were normalized using DESeq2 (Version: DESeq2_1.34.0) median of ratios method using R package (Version: R4.0.4.) and were used for downstream targeted gene expression statistical analysis.

### Flow cytometry analysis

50 µl fresh EDTA whole blood and antibody solution were mixed by vortex for 4 s, and followed by 10 min incubation in room temperature avoiding light. Afterwards 1 ml NH_4_Cl lysing solution (BD Pharm lyse, BD Biosciences) were added to the stained sample, and vortexed for 5 s. Then the solution was incubated at room temperature for 10 min without light. 300 µl of the lysed sample was used for flow cytometry measurement (Beckman Coulter). The antibody solution contained 5 antibodies, including IGF-1R (APC, Life Technologies), CD14 (FITC, Agilent technologies), CD16 (PE, eBioscience), CD45 (PECy7, Beckman Coulter), HLA-DR (PB, Beckman Coulter). Monocytes were first gated by forward scatter and side scatter, and then by CD45. The geometric mean of IGF-1R was used for t-test analysis.

## Results

### The effect of urate on the expression of *IGF1R*

**Figure 1:**
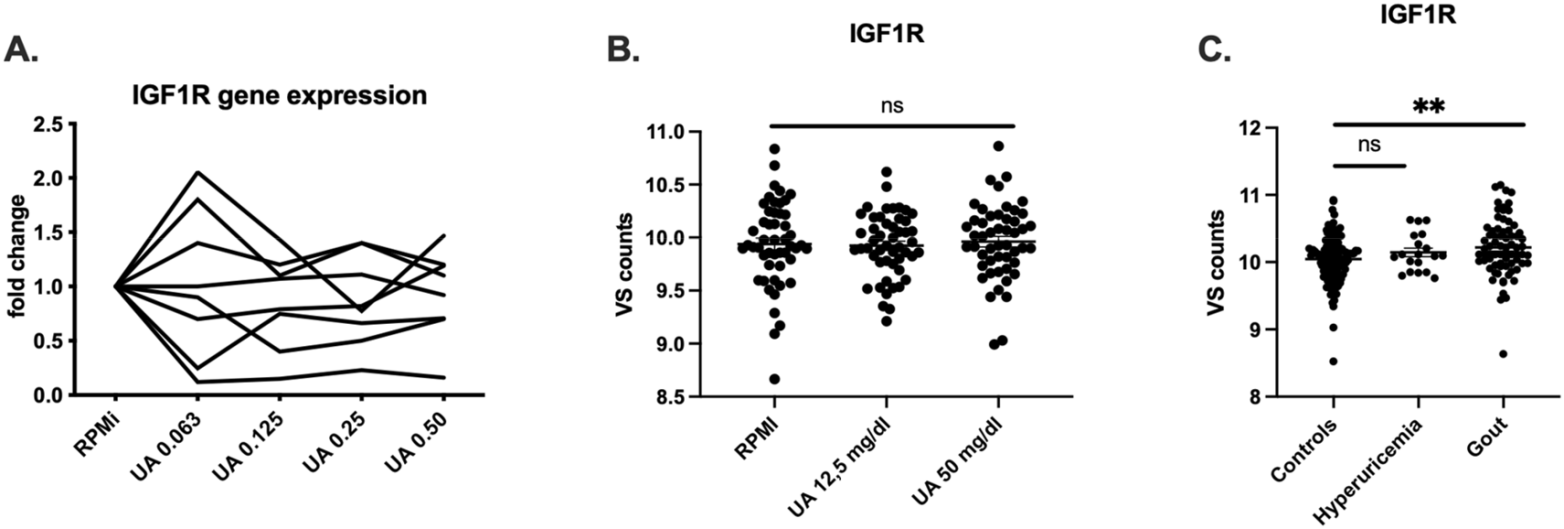
Urate effects on mRNA expression in vitro and basal expression of *IGF1R* of the studied groups. (**A**) mRNA expression of freshly isolated PBMCs originating from healthy donors (n = 8) treated with different concentrations of uric acid (UA). (**B**) mRNA expression of freshly isolated PBMCs originating from gout patients (n = 50) treated with UA for 24 h and restimulated with LPS 10 ng/ml. Repeated measurements one-way ANOVA and Tukey's multiple comparisons test, *p < 0.05. (**C**) mRNA expression of freshly isolated PBMCs originating from healthy controls (n = 113), hyperuricemic patients (n = 19) and gout patients (n = 72). The data is represented as normalized counts. Brown-Forsythe and Welch ANOVA, *p < 0.05.

To assess the effect of urate on the expression of *IGF1R*, mRNA was measured after stimulation of PBMCs of eight healthy donors with different concentrations of urate for 24 h. No differences were observed in the expression of the receptor itself (Fig. 1A). In addition, we assessed the mRNA level of the receptor in PBMCs from gout patients in the experimental setup of 24 h urate priming followed by stimulation with LPS 10 ng/ml (TLR4 ligand) for another 24 h. In line with the previous results, no differences regarding expression of *IGF1R* were observed after stimulation of the cells (Fig. 1B). In contrast, we could observe an increased steady-state mRNA expression of *IGF1R* in unstimulated monocytes originating from gout patients compared to normouricemic controls (Fig. 1C).

### Activation or inhibition of IGF1R does not modify cytokine production in urate priming or trained immunity experiments

**Figure 2:**
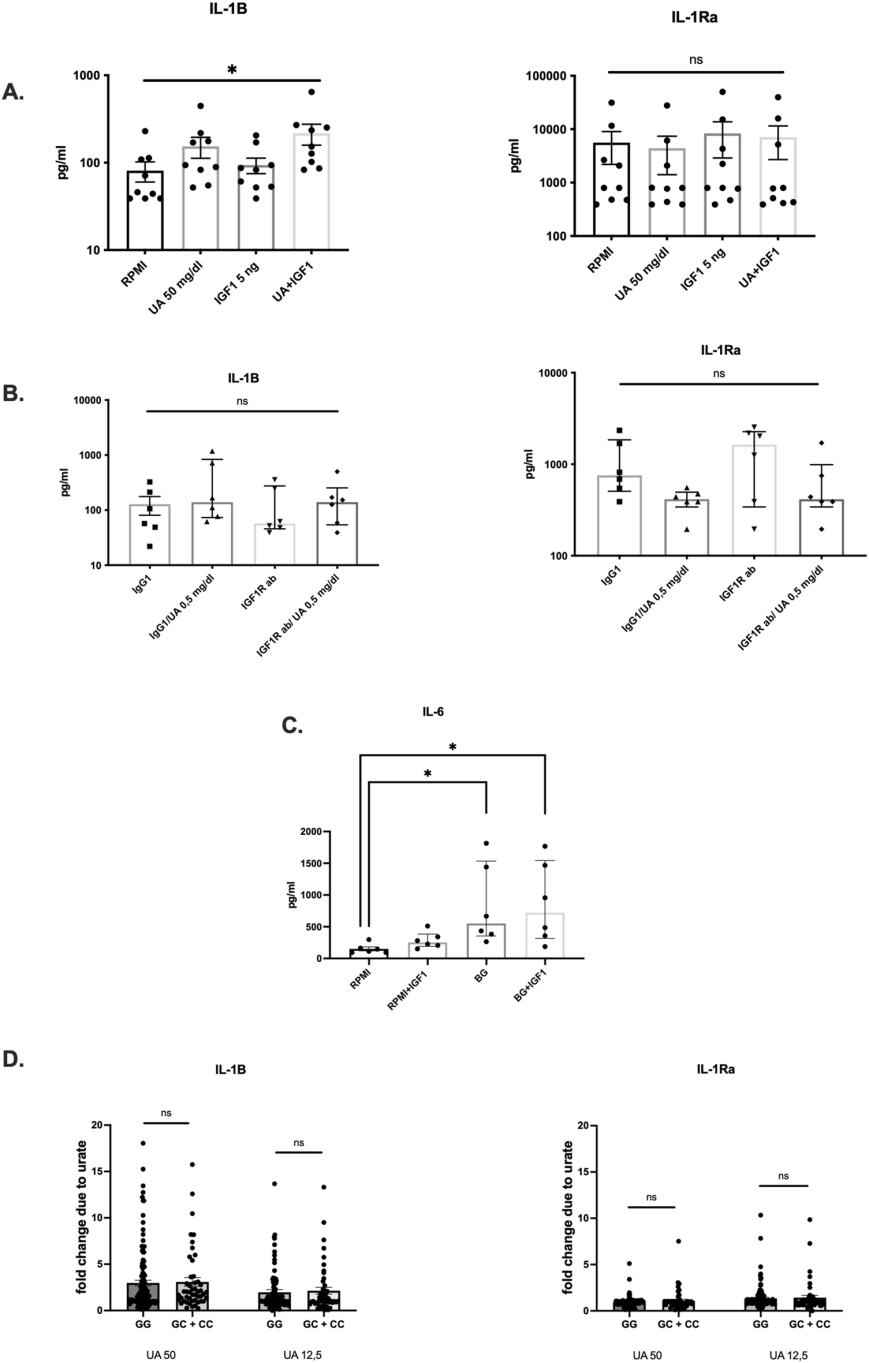
Role of IGF1R in urate induced inflammation in vitro. (**A**) Freshly isolated PBMCs isolated from healthy controls (n = 9) were stimulated with RPMI, uric acid 50 mg/dl, IGF-1 recombinant protein (R&D Systems) 5 ng for 24 h. After 24 h the cells were restimulated with LPS 10 ng/ml together with MSU 300 mg/dl. (**B**) Freshly isolated Percoll enriched Monocytes (n = 6) were treated with RPMI, IGF1R antibody (R&D Systems) or IgG1 isotype control and uric acid 50 mg/dl for 24 h. After 24 h monocytes were restimulated with LPS 10 ng/ml with MSU 300 mg/dl. (**C**) Freshly isolated PBMCs (n = 6) were trained in vitro with IGF-1 5 ng and beta-glucan (BG) 1 μg/ml for 24 h, subsequently washed, rested for 5 days, and at day 6 restimulated for 24 h with 10 ng/ml LPS. (**D**) Freshly isolated PBMCs originating from healthy controls (n = 174). Concentration IL-1β and IL-6 measured in the supernatants of PBMCs after stimulation with urate of conc. 50 mg/dl and 12.5 mg/dl for 24 h, followed by restimulation with LPS 10 ng/ml. IL-1β, and IL-1Ra (R&D Systems, Minneapolis) was measured in supernatant by ELISA. Graphs depict means ± SEM. Friedman test, Dunn's multiple comparisons test, p* < 0.05.

Next, we studied the possible contribution of IGF1R to inflammation in an experimental setup consisting of 24 h exposure to recombinant IGF-1 protein (Fig. 2A) (R&D Systems) or anti-IGF1R neutralizing antibody (Fig. 2B) in the presence or absence of solubilized urate, followed by 24 h stimulation with LPS and MSU crystals. Cells treated with solubilised urate (50 mg/dl) together with the recombinant IGF-1 protein (5 ng) produced more IL-1β, but not IL-6 or IL-1Ra compared to control. The cytokine production was not modified by anti-IGF1R neutralizing antibody. Further, we tested if IGF-1 could influence cytokine production in a trained immunity experimental design^29,30^, consisting of stimulation for 24 h with IGF-1 or β-glucan (BG) as positive control, followed by washout and rest for 5 days and subsequent second stimulation with LPS (10 ng/ml). We did not observe an enhanced training effect by IGF1 (Fig. 2C). Bekkering et al*.* reported SNPs in *IGF1R* (rs150571637, rs9672558, rs34428109, rs1573891) associated with trained immunity in response to Bacille Camette-Guerin (BCG) and beta-glucan isolated from *C. albicans*^31^. Thereafter we assessed *IGF1R* rs1573891 in our study and found no association of this SNP to priming of PBMCs by soluble urate (Fig. 2D).

### *IGF1R* rs6598541 SNP and *IGF1R* expression levels in freshly isolated PBMCs

**Figure 3:**
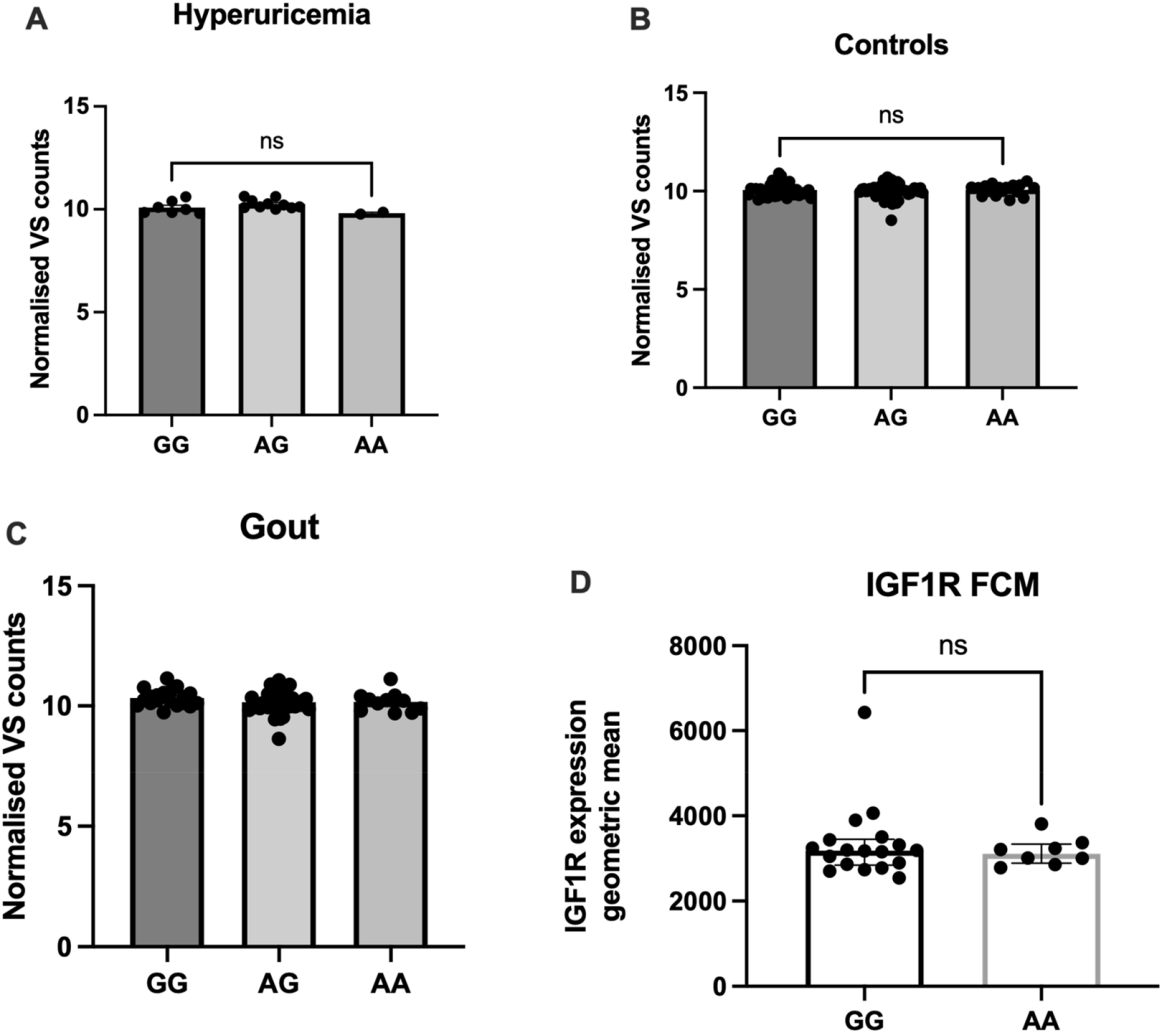
Corellation of the GWAS SNP rs6598541 with IGF1R expression levels. mRNA expression of freshly isolated PBMCs originating from (**A**) hyperuricemic controls (n = 19) (**B**) normouricemic controls (n = 105) and (**C**) gout patients (n = 68). The data is represented as normalized counts. (**D**) IGF1R protein surface expression measurment by flow cytometric assay of unstimulated PBMCs originating from healthy donors (n = 26). Brown-Forsythe and Welch ANOVA, *p < 0.05.

We further examined the *IGF1R* SNP rs6598541, previously associated with serum urate levels^21,23^ and gout^24^, for association with *IGF1R* expression in patients with gout, hyperuricemia and normouricemic controls. The basal expression level of *IGF1R* in freshly isolated PBMCs from the three groups (Fig. 3A–C) was not associated with rs6598541. Additionally, flow cytometric assessment of IGF1R surface expression on unstimulated PBMCs from healthy donors was also not association with *IGF1R* rs6598541 (Fig. 3D).

### *IGF1R* rs6598541 SNP and cytokine production in stimulated PBMCS

**Figure 4:**
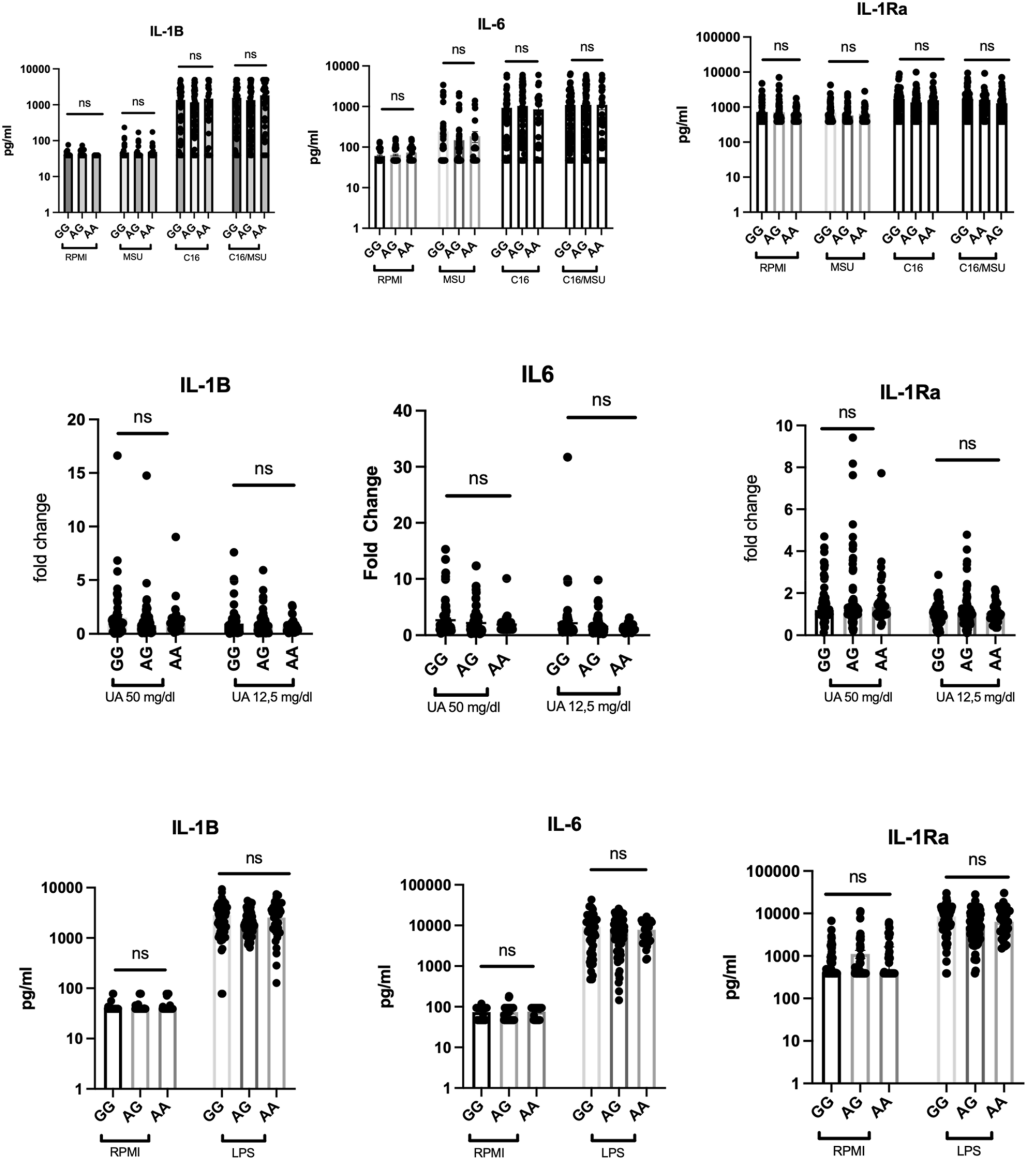
Correlation of the rs6598541 SNP with ex vivo cytokine production. (**A**) Freshly isolated PBMCs originating from healthy controls (n = 187) stimulated with RPMI, MSU 300 mg/dl, C16, C16/MSU 300 mg/dl for 24 h. After 24 h the supernatants were collected and IL-1β, IL-6 and IL-1Ra (R&D Systems, Minneapolis) was measured. (**B**) Concentration IL-1β, IL-6 and IL-1Ra measured in the supernatants of PBMCs after stimulation with uric acid of conc. 50 mg/dl and 12.5 mg/dl for 24 h, followed by restimulation with LPS 10 ng/ml. (**C**) IL-1β, IL-6 and IL-1Ra levels measured in the supernatants of PBMCs after stimulation with LPS 100 ng for 24 h. Graphs depict means ± SEM.

To further study the possible implication of rs6598541 on inflammation, we assessed the ex vivo cytokine secretion by freshly isolated PBMCs challenged with various stimuli in association with the *IGF1R* rs6598541 genotypes: cytokine production in response to 24 h stimulation with MSU, palmitate (C16.0) or the combination of MSU crystal with C16.0 (Fig. 4A); urate priming for 24 h and stimulation with LPS for 24 h (Fig. 4B); or 24 h stimulation with LPS 100 ng (Fig. 4C). No significant association was found between the SNP and ex vivo cytokine production (IL-6, IL-1β or IL-1Ra). The same analysis of data obtained following the same experiment carried out for the hyperuricemia individuals and for patients with gout(both active and non-active form) also showed no significant differences for these conditions between genotypes (supplementary Figs. 1, 2, 3).

## Discussion

IGF-1 is known to have modulatory roles in human immune responses and a regulatory role in the activation of the peripheral monocytes^26,32^. Interestingly, IGF-1 has dual roles, being involved in inflammation in a context-dependent mode, presenting both pro- and anti-inflammatory properties. After myocardial infarction, IGF-1 acted like an anti-inflammatory cytokine on myeloid cells in vitro while negating the pro-inflammatory phenotype of neutrophils and macrophages in vivo^33^. Also, several in vitro studies revealed anti-inflammatory properties of IGF-1 on astrocytes and microglia^34,35^, whereas another study focused on it’s role in decreasing the release of IL-1Ra and increasing IL-1β^36^.

The IGF-1 pathway has recently been shown to be involved in the induction of trained immunity. Since innate immune memory is increasingly shown to be important for urate-induced inflammation, in the present study, we were interested in assessing IGF-1/IGF1R signaling in urate priming using primary PBMCs.

Our data indicate that IGF-1 does not contribute to urate-induced inflammation and blocking IGF1R did not influence the inflammatory responses triggered by urate. We show that urate does not modulate the expression of *IGF1R* itself in vitro in PBMCs from healthy donors nor in vivo in PBMCs from gout patients.

However, we noticed an increased steady-state mRNA expression of *IGF1R* in unstimulated PBMCs from gout patients. Increased expression of *IGF1R* was also seen in leukocytes of rheumatoid arthritis patients, which was associated with systemic inflammation and pain^37^. Interestingly, RA patients with high *IGF1R* gene expression values were found to have low IGF-1 serum concentrations^37^. In contrast, elevated circulating IGF-1 concentrations were reported in patients with gout and insulin resistance^38^.

Bekkering et al*.* describe four SNPs in *IGF1R* associated with differential cytokine production in monocytes trained with β-glucan or BCG vaccine (Bacillus Calmette–Guérin)^25^. We have also assessed one of these four SNPs with respect to urate priming and found no association of these variants to changes in cytokine profiles after urate exposure (Fig. 2D). Therefore, our data provide no evidence for a role of IGF1R in urate-mediated inflammatory priming.

We explored association of the *IGF1R* rs6598541 SNP, identified as a genetic susceptibility variant for gout and serum urate levels^21,23,24^, with cytokine production in human PBMCs. *IGF1R* genetic variants are potentially functionally relevant in gout and hyperuricemia since the genetic control of urate levels and risk of gout at the *IGF1R* locus also colocalizes with genetic control of *IGF1R* expression data^23^. For *IGF1R* rs6598541, the gout risk and elevated serum urate associated allele A is associated with lower *IGF1R* expression in heart tissue (left ventricle)^39^. In our study, when assessing freshly isolated PBMCs from patients with gout or controls we did not observe association of *IGF1R* gene expression with rs6598541. In addition, IGF1R surface expression evaluated by flow cytometry in monocytes of healthy donors also did not show modified IGF1R expression associated with rs6598541 genotype. In line with this, ex vivo cytokine secretion by freshly isolated PBMCs challenged with certain stimuli was also not associated with rs6598541. This indicates that this SNP may exert relevant functional roles in gouty inflammation, but they may be tissue specific and mononuclear cells do not show *IGF1R* expression patterns nor inflammatory cytokine production capacity in relationship to this SNP.

However, data on the role of IGF-1 on urate control give more mechanistic insight. A genetic variant associated with elevated IGF-1 levels (rs35767) correlated to diminished serum urate levels and higher uricosuria^40^. Exposure of HEK293 human embryonic kidney cells to IGF1 resulted in a dose-dependent increase of secretory urate transporters MRP4, NPT1 and ABCG2 and a simultaneous reduction of *GLUT9* expression at both the mRNA and protein levels^40^. GLUT9a is the chief transporter for basolateral exit of reabsorbed urate into blood^41^. As reported by Kottgen et al., the urate-associated *IGF1R* rs6598541 variant is also associated to lower fractional excretion of urate, supporting a role of the IGF1-IGF1R axis on urate transport. This is supported by data showing that, in *Xenopus laevis* oocytes expressing human IGF1R and urate transporters, IGF-1 promotes urate uptake via IGF1R^39^. Therefore *IGF1R* association to gout is most probably exerted via urate control rather than inflammatory mechanisms, via IGF1R, *GLUT9* expression and activation, leading to urate reabsorbtion^42^.

Another mechanism linking *IGF1R* rs6598541 to urate levels is insulin resistance. *IGF1R* rs6598541 was reported to be genome-wide associated with fasting glucose: people without diabetes carrying the A-allele have increased fasting glucose levels, suggesting an association with insulin resistance^43,44^. It is known that hyperuricemia might be contributed to the effect of insulin on decreasing renal urate clearance and sodium excretion in individuals^42,45,46^.

In conclusion, our data do not support a role of IGF-1/IGF1R signaling in soluble urate-induced inflammation in primary PBMCs. Consistent with this, functional data associated to genetic variants in *IGF1R* shows no association with ex vivo* c*ytokine production.

Therefore, these results suggest that a role of IGF1R in gout may be more relevant for the control of urate levels rather than on the inflammatory process.

### Supplementary Information

Supplementary Figures.

Supplementary Tables.

## Data Availability

All the datasets generated and analysed during the current study are available in the Supplementary Table 1.
