# Supplementary material for: GWAS-identified hyperuricemia-associated IGF1R variant rs6598541 has a limited role in urate mediated inflammation in human mononuclear cells
Source: Sci Rep. 2024 Feb 12;14:3565. doi: 10.1038/s41598-024-53209-7 (PMC10861580; doi:10.1038/s41598-024-53209-7)
Supplement: Supplementary file 1 — Supplementary Figures. [file 41598_2024_53209_MOESM1_ESM.docx]

**
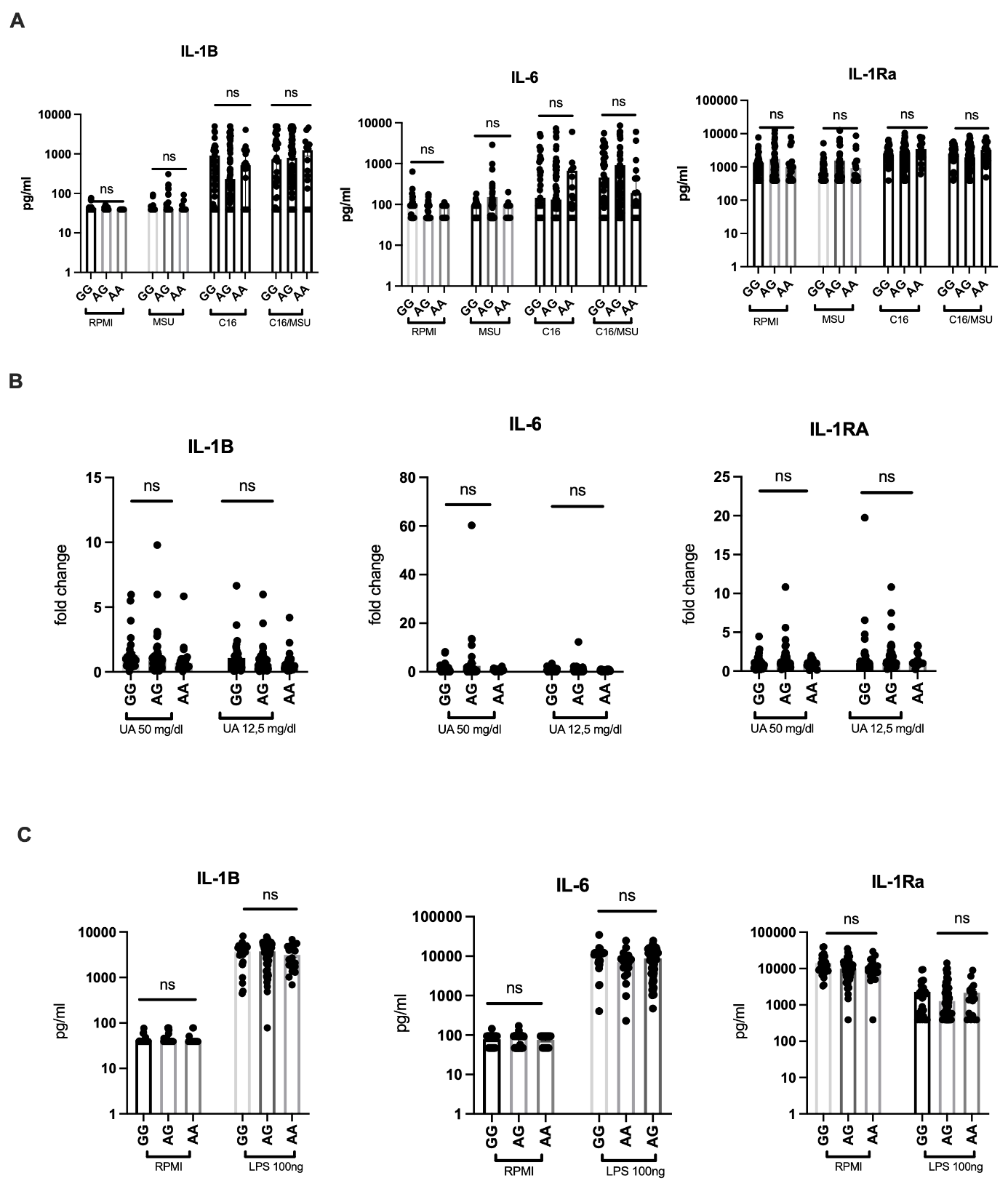
**

**Suppl Figure 1.** A.Freshly isolated PBMCs originating from **gout patients** (n=119) stimulated with RPMI, MSU 300mg/dl, C16, C16/MSU 300mg/dl for 24h. After 24h the supernatants were collected and IL-1β, IL-6 and IL-1Ra (R&D Systems, Minneapolis) was measured. **B**. Concentration IL-1β, IL-6 and IL-1Ra measured in the supernatants of PBMCs after stimulation with uric acid of conc. 50mg/dl and 12,5 mg/dl for 24h, followed by restimulation with LPS 10 ng/mL. **C.** Concentration IL-1β, IL-6 and IL-1Ra measured in the supernatants of PBMCs after stimulation with LPS 100ng for 24h. The lowest range of detection was 78 pg/ml for IL-1β; 390 pg/ml for IL-1Ra and 188 pg/ml for IL-6. Graphs depict means+/−SEM.


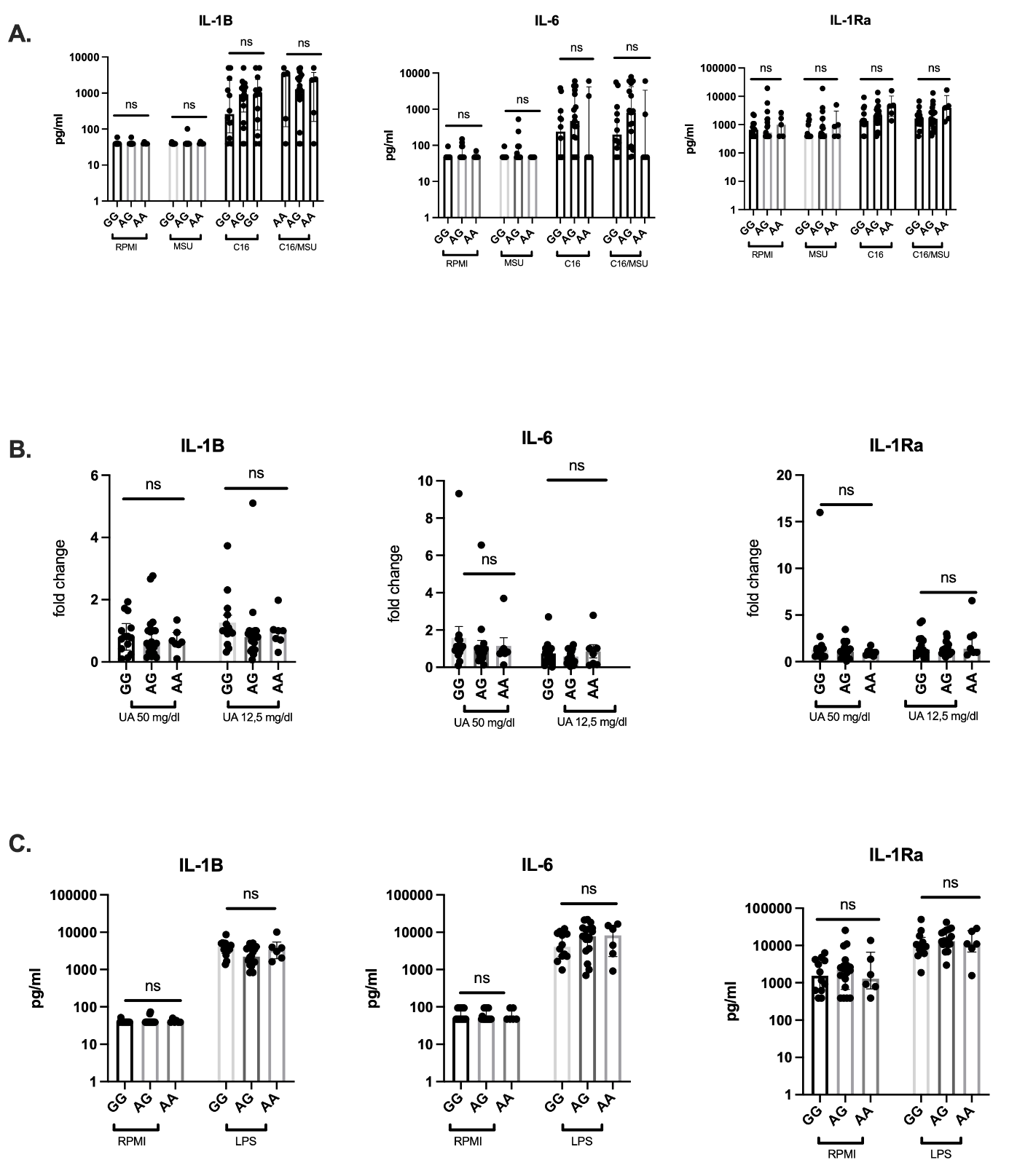


**Suppl Figure 2.** A.Freshly isolated PBMCs originating from **hyperuricemic patients** (n=40) stimulated with RPMI, MSU 300mg/dl, C16, C16/MSU 300mg/dl for 24h. After 24h the supernatants were collected and IL-1β, IL6 and IL1Ra (R&D Systems, Minneapolis) was measured. **B**. Concentration IL-1β, IL-6 and IL-1Ra measured in the supernatants of PBMCs after stimulation with uric acid of conc. 50mg/dl and 12,5 mg/dl for 24h, followed by restimulation with LPS 10 ng/mL **C.** Concentration IL-1β, IL-6 and IL-1Ra measured in the supernatants of PBMCs after stimulation with LPS 100ng for 24h. The lowest range of detection was 78 pg/ml for IL-1β; 390 pg/ml for IL-1Ra and 188 pg/ml for IL-6. Graphs depict means+/−SEM.


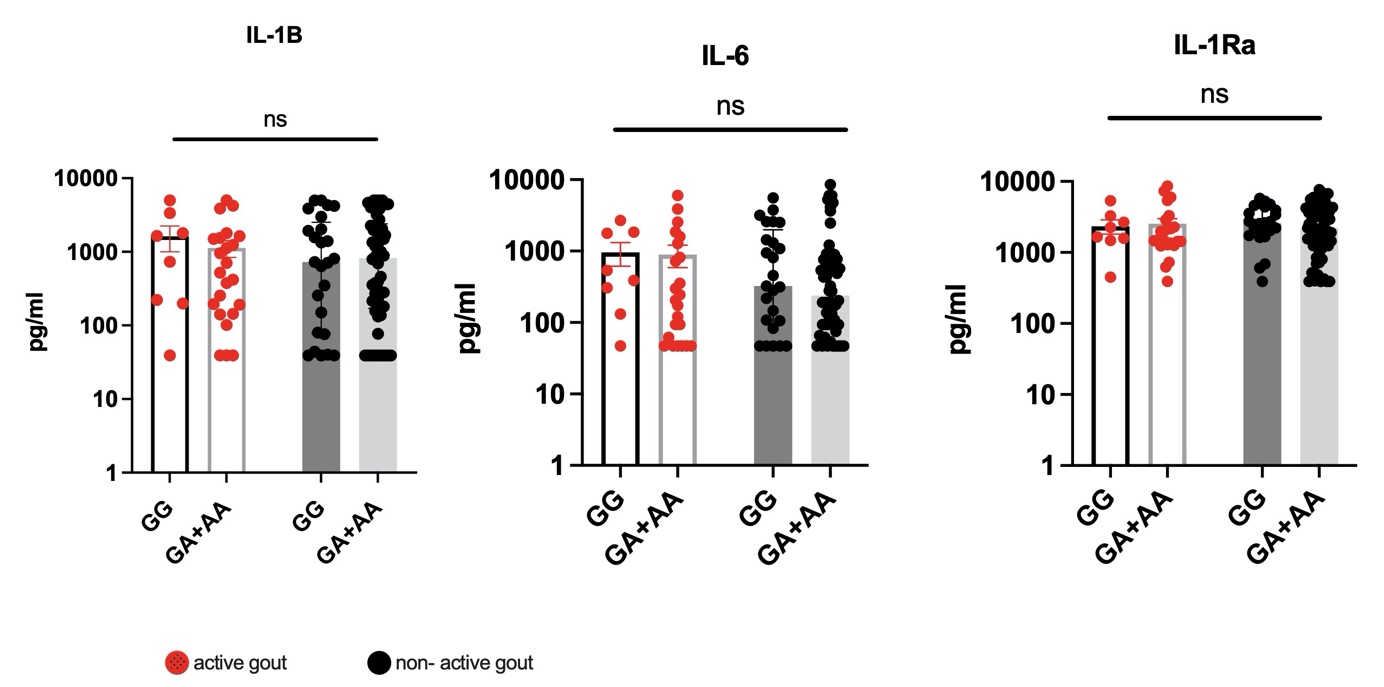


**Suppl Figure 3.** A.Freshly isolated PBMCs originating from **gout patients** (n=114 from which n=31 were patients with active gout,marked with red) were stimulated with C16/MSU 300mg/dl for 24h. After 24h the supernatants were collected and IL-1β, IL-6 and IL-1Ra (R&D Systems, Minneapolis) was measured. Graphs depict means+/−SEM.
